# Supplementary material for: Employing foreign caregivers: A qualitative study of the perspectives of older stroke survivors
Source: PLoS One. 2025 Jan 3;20(1):e0316757. doi: 10.1371/journal.pone.0316757 (PMC11698342; doi:10.1371/journal.pone.0316757)
Supplement: S1 Table — (DOCX) [file pone.0316757.s002.docx]

**S2 Table. Interview Guide**

| **Interview questions** | **Prompt** |
| --- | --- |
| **Part 1: Context** | |
| 1. Please describe the caregiver’s role in your household. | - What led you to decide to hire a foreign caregiver? - How many days a week does she work, and how many hours does she typically work each day? - How would you evaluate her performance so far? - How would you assess your caregiver’s knowledge of stroke care and management? - Has your caregiver received any stroke-related or medical training? - In your opinion, is formal caregiver training necessary for this role? Why or why not? |
| 1. We’d like to know more about your relationship with your caregiver. How would you describe your overall relationship? | - How would you characterize your relationship with your caregiver? - Have you encountered any challenges in your relationship with your caregiver? If so, what were those challenges? - Can you describe a specific situation where you faced difficulties in your relationship? How did you handle or resolve the situation? - How do you and your caregiver manage communication and address misunderstandings and conflicts? |
| **Part 2: Expectations** | |
| 1. What did you initially expect of this caregiver before hiring her? | - From your perspective, what do you believe are your caregiver’s duties and responsibilities? - Is there anything specific you believe your caregiver should do to better support you in your daily life? - What are your personal expectations of her as a caregiver? |
| **Part 3: Experiences with Caregiving—Benefits, Challenges, and Cultural Perspectives** | |
| 1. What positive experiences or benefits have you noticed from receiving care from your caregiver? | - Have you noticed any positive changes in your life since employing this caregiver? If so, can you provide specific examples? - Do you believe her assistance has contributed to your recovery progress? In what specific ways has she had an effect? |
| 1. Have you faced any challenges while receiving care from your caregiver? If so, please describe them. | - Have you encountered any unexpected challenges since employing your caregiver? If so, can you provide specific examples? - Has the presence of your caregiver affected your life or your family’s life in any significant way? If so, how? - Have you ever felt stressed or uncomfortable with your caregiver? If so, how has this stress affected your emotional or physical wellbeing? - Does the cost of the caregiver’s salary put a strain on you or your family’s financial situation? If so, how? |
| 1. Have you noticed any cultural differences between yourself and your caregiver? If so, how have these differences affected the caregiving experience? | - Have you experienced any cultural differences while working with your caregiver? If so, what were they? - In your opinion, do these cultural differences affect her ability to perform her caregiving duties? If so, how and why? - Can you provide an example of how cultural differences have influenced your interactions or her approach to caregiving? |
| 1. Is there anything else you would like to share about your experience working with your caregiver that we haven’t covered? |  |
