# Supplementary material for: Employing foreign caregivers: A qualitative study of the perspectives of older stroke survivors
Source: PLoS One. 2025 Jan 3;20(1):e0316757. doi: 10.1371/journal.pone.0316757 (PMC11698342; doi:10.1371/journal.pone.0316757)
Supplement: S2 Table — (DOCX) [file pone.0316757.s003.docx]

**S3 Table. Sample quotes**

| **Themes** | **Subthemes** | **Quotes** |
| --- | --- | --- |
| **Theme 1. Motivations for Hiring a Foreign Caregiver** | Need for supervision and physical assistance | *“The doctor said that my hip joints and knees were broken and that my condition would worsen. Once my condition worsened, the doctor said that I should hire a caregiver. After choosing a caregiver, more than 2 years passed, and I suffered a stroke.”* (PS28) |
|  | Lack of family caregiving support | *“Everyone at home is too busy to look after me, so I hired an assistant.”* (PS20) |
|  | Financial limitations | *“Hiring a caregiver is expensive. My family spends at least $NT50,000 per month on me. This covers the rent, my foreign caregiver’s salary, and food. My children work very hard.”* (PS01) |
| **Theme 2. Expectations of Participants Toward the Foreign Caregivers** | Being obedient and submissive | *“I want her to listen to me. She needs to pay attention to what I'm saying, you know?”* (PS02)  *“As long as she behaves and doesn’t run around, everything’s fine.”* (PS05) |
|  | Learning Mandarin and embracing Taiwanese culture | *“She should be able to speak Chinese. Taiwanese is fine, too.”* (PS02) |
|  | Mastering stroke care and homemaking | *“She should be familiar with using certain equipment and assisting with moving me around, you know? Basic stuff.”* (PS17)  *“The most important thing is to take good care of me and make sure I don’t fall. That’s crucial.”* (PS09)  *“It’s just household chores. She should definitely do things like washing clothes, mopping the floor, and taking out the trash. These tasks are all part of her job.”* (PS11)  *“She needs to understand my dietary needs, what I can and can’t eat. That way, she can prepare meals that suit me.”* (PS21) |
|  | Providing care for other family members | *“When my son comes home, shouldn’t she be cooking for him? Does she expect my son to eat out instead?”* (PS01)  *“Sometimes we’re all busy or not at home, so she also needs to help take care of my 2-year-old granddaughter. Otherwise, there’s no one to look after her.”* (PS11)  *“Besides taking care of me, she can also help take care of my mom.”* (PS26) |
| **Theme 3. Challenges Related to Employing a Foreign Caregiver** | Communication difficulties | *“It’s like, if we speak Taiwanese, she might think we’re talking smack about her, you know? Then she’ll give us that stink-eye.”* (PS01)  *“Linda accompanies me to rehab outpatient appointments. The doctor reminds me of important things, but because Linda doesn’t speak Chinese very well, she can’t help me remember the doctor’s instructions.”* (PS04)  *“When we tell her what to do, she doesn’t understand, she doesn’t know. We have to guess everything. We’d ask her whether she knows how to do something, and she’d say she does, but actually she doesn’t.”* (PS11) |
|  | Cultural differences | *“They’re used to not showering at night, I can’t stand it; it’s so unhygienic!”* (PS02)  *“Culturally, they’re Muslims, they don’t eat pork. It’s something we find hard to accept because we’re used to eating pork at home. It’s really inconvenient.”* (PS05)  *“Being Muslim, they don’t eat pork, but Taiwanese people love pork. This becomes a hassle when we take her out to eat.”* (PS27) |
|  | Caregiving skill deficiencies | *“For example, she has never been trained in tasks like inserting tubes for patients or assisting patients with rehabilitation walking. She’s completely inexperienced in these areas.”* (PS25) |
|  | Concerns with foreign caregivers’ attitudes and behaviors | *“I have to call her four or five times before she responds. When it’s time to get up, I call her but she doesn’t respond. I can call dozens of times and still not get a response. If I raise my voice at her, she gets upset. If I call louder, she just throws a tantrum. She ignores me.”* (PS02)  *“She goes out with my wife to push me in the wheelchair, but she always complains about it being too much trouble. She gets annoyed after just a few pushes. If I ask her to push a little further, she throws a tantrum and refuses to push anymore!”* (PS15)  *“Indonesians are all like that. She wakes up in the morning and just keeps talking nonstop, on the phone, chatting, you name it, she just keeps going for ages. She’s not even focused when she’s working, just keeps chatting away.”* (PS23)  *“She’s on the phone even when she’s working. She’s not focused at all.”* (PS26)  *“It seems like there’s something going on between her and my husband. Feelings may have developed over time. I’m serious.”* (PS14) |
