# Supplementary material for: Employing foreign caregivers: A qualitative study of the perspectives of older stroke survivors
Source: PLoS One. 2025 Jan 3;20(1):e0316757. doi: 10.1371/journal.pone.0316757 (PMC11698342; doi:10.1371/journal.pone.0316757)
Supplement: Attachment — Submitted filename: Response to Reviewers 2024.10.17.docx [file pone.0316757.s004.docx]

**Response to Reviewers**

**Comments to the Author**

1. Is the manuscript technically sound, and do the data support the conclusions?

The manuscript must describe a technically sound piece of scientific research with data that supports the conclusions. Experiments must have been conducted rigorously, with appropriate controls, replication, and sample sizes. The conclusions must be drawn appropriately based on the data presented.

Reviewer #1: Yes

Reviewer #2: No

2. Has the statistical analysis been performed appropriately and rigorously?

Reviewer #1: N/A

Reviewer #2: N/A

3. Have the authors made all data underlying the findings in their manuscript fully available?

The [PLOS Data policy](http://c05y1x9s.r.us-east-2.awstrack.me/L0/http:%2F%2Fwww.plosone.org%2Fstatic%2Fpolicies.action%23sharing/1/010f019222ac9a98-89135d1f-7ce8-44ab-9029-1a2661981883-000000/FD0Tc5dCyOvll-XzsF_uOxFQNow=177) requires authors to make all data underlying the findings described in their manuscript fully available without restriction, with rare exception (please refer to the Data Availability Statement in the manuscript PDF file). The data should be provided as part of the manuscript or its supporting information, or deposited to a public repository. For example, in addition to summary statistics, the data points behind means, medians and variance measures should be available. If there are restrictions on publicly sharing data—e.g. participant privacy or use of data from a third party—those must be specified.

Reviewer #1: No

Reviewer #2: No

**Response:** We are unable to share the data (interview transcripts) due to the significant amount of personal and sensitive information they contain about the participants. Moreover, the informed consent form assured the participants of the privacy and confidentiality of their interviews, which are protected under the oversight of the Office of Human Research at Taipei Medical University. For further inquiries, please contact the Office of Human Research at ohr@tmu.edu.tw.

4. Is the manuscript presented in an intelligible fashion and written in standard English?

PLOS ONE does not copyedit accepted manuscripts, so the language in submitted articles must be clear, correct, and unambiguous. Any typographical or grammatical errors should be corrected at revision, so please note any specific errors here.

Reviewer #1: Yes

Reviewer #2: No

**Response:** We sincerely appreciate the valuable feedback provided by both reviewers, which has greatly improved the quality of our manuscript. We have carefully considered and addressed all of your suggestions and concerns in this revision, and we believe the changes have strengthened the clarity and rigor of our work. Please see our itemized responses to each comment below.

5. Review Comments to the Author

Please use the space provided to explain your answers to the questions above. You may also include additional comments for the author, including concerns about dual publication, research ethics, or publication ethics. (Please upload your review as an attachment if it exceeds 20,000 characters)

**Reviewer #1:** Review ”Employing foreign caregivers: exploring perspectives of older stroke survivors – a qualitative study”

Thank you for the opportunity to review this manuscript with, from my point of view, a problematic topic. The authors themselves mention exploitation and I become very badly affected when reading the results. The expectations of the persons affected by stroke widely exceeds what is reasonable to expect. How can anyone consider it to be ok to expect persons to work 24 hours per day as is stated in line 250? This represent a very dehumanized way of looking at other persons. The researchers make a good discussion of this very problematic issue.

1. Abstract
   In the section for Results, the themes are given, however, not with the correct names. Make sure to use the correct names from the results or re-write the text in the abstract so it is evident that what is written there is not the actual themes, but a summary of what they tell about.

**Response:** Thank you for pointing out this inconsistency. We have carefully revised the Results section in the abstract to ensure that the theme titles now accurately align with those used in the main text (see Page 3 / Line 47 – 51).

2. Introduction
   A little more about the health care system in Taiwan needs to be described for an international audience to be able to understand the article. Which care services are available for persons who are in need of daily care, but not needing hospital care? What is the cost for such services for the individual person in need?

**RESPONSE:** We added the following background: “Since 1992, the Taiwanese government has introduced live-in foreign caregivers under the regulation titled “Temporary Measure to Address Shortages of Manpower for Providing Care for Households’ Disabled,” in response to the needs of families with disabled members, addressing the crisis of the local caregiver shortage [12]. Although Taiwan’s Ten-Years Long-Term Care Project (LTC) offers various home-based and community-based services, such as adult daycare and home care provided by Taiwanese care workers [13], many individuals with care needs still prefer to hire foreign caregivers. By 2019, approximately 28% of people with disabilities were cared for by live-in foreign caregivers [14]. In 2021, 225,432 foreign caregivers were employed by families to provide care services, compared to only 74,601 Taiwanese care workers [15]. This gap may be attributed to the advantage of live-in foreign caregivers, who offer 24-hour assistance at an affordable cost (USD 550 to 650, per month)[16, 17].”

(see Page 4 - 5, Line 79 – 90)

3. On p 11, lines 86-87, it is stated that “a” scoping review has highlighted but then two references are given. When using two, the word “a” is not sufficient. However, reference 16 does not seem to be a scooping review.

**RESPONSE:** Thank you for your comment. We have addressed this by removing the redundant reference and now only cite the appropriate scoping review: Salami B, Duggleby W, Rajani F. The perspective of employers/families and care recipients of migrant live-in caregivers: a scoping review. Health & Social Care in the Community. 2017;25(6):1667-78. (see Page 6, Line 99)

4. On p 11, lines 87-90, a statement is given about existing qualitative investigations, where the scoping review is given as one of the references. Are the authors sure that all studies included in the scoping review are performed with qualitative methods?

**RESPONSE:** We apologize for the confusion. The cited scoping review included a variety of international studies that explored the experiences of families and live-in caregivers; it was not limited to qualitative research. Our intention was to emphasize that the review identified a lack of research in Asia on care recipients' experiences with foreign caregivers, regardless of whether the studies were qualitative or quantitative (while this review did review many qualitative studies regarding the experiences of families and live-in caregivers). To clarify, we have revised this section as follows: “A scoping review of international literature on the experiences of families and live-in caregivers highlighted the lack of research conducted in Asia regarding care recipients' experiences with the care they receive from foreign caregivers [21]. In fact, there is limited literature exploring the perspectives of individuals with care needs and their families on hiring foreign caregivers. Among these studies, some qualitative investigations have shed light on the complex and interdependent relationships between foreign caregivers and their care recipients [21-23].”

(see Page 6, Line 99 – 105)

5. Method
   Setting and participants
   It is unclear to me if all patients with a previous diagnosis of stroke are scheduled for outpatient visits at the physical medicine and rehabilitation departments of the included hospitals or if the patients who are scheduled for such visits constitute a special selection in some way? I would not expect that everyone who has got a diagnosis of stroke and live in the catchment area of one of the included hospitals, would still be scheduled for outpatient visits at these departments more than five years after their stroke. This needs to be clarified in order to understand if the available participants in any way constitutes a selected group of patients.

**RESPONSE:** Since Taiwan’s National Health Insurance (NHI) covers all citizens, the out-of-pocket costs for rehabilitation services are minimal. As a result, patients face little financial burden when continuing outpatient rehabilitation after being discharged from the hospital. Additionally, many hospitals do not impose limits on the duration of outpatient rehabilitation programs, even if providers determine that the program is no longer beneficial for the patient [4]. To avoid confusion, we added the following description in the Study Setting and Recruitment: “Under Taiwan’s National Health Insurance (NHI) system, there are no restrictions on the use of outpatient rehabilitation services for stroke patients, allowing us to recruit patients at various stages of recovery.” (see Page7, Lines 130 – 133)

6. Procedure
   On p. 13 the development of the semi-structured interview guide is described. I would suggest to also give the number of questions, not only state that there were “various questions” together with the examples tat are given.

**RESPONSE:** Thank you for this suggestion. We added more information about the interview guide as following: “This guide included seven major open-ended questions, which were reviewed by an interdisciplinary expert panel, as well as stroke survivors, and caregivers. The full interview guide is available in **S2 Table**, with examples of key questions provided below…” (see Page 9 – 10, Lines 173 – 176)

7. On p 14, line 140, present tense is used, while past tense should be used, on for example “family caregivers are encouraged”.

**RESPONSE:** We apologize for the error. We revised this sentence to “Family caregivers were encouraged…” (see Page 10, Lines 185)

8. Data analysis
   On p 12, line 102 the study is stated to employ a descriptive phenomenological approach, but in the section for Data analysis, p 14, line 149, it is stated that a thematic analysis was performed. These two statements are not compatible and cannot both be true Please revise this. I cannot find any signs in the text, of this study being a phenomenological study so I would suggest to keep the claim about the thematic analysis and omit the phenomenological claims. This also gives a need to revise the aim. When a phenomenological analysis has not been performed, the results is not “lived experience”.

**RESPONSE:** Thank you for your feedback. Based on a thorough literature review, the nature of our results, and the reviewer's suggestions, we have revised the study's design from a phenomenological approach to a descriptive qualitative approach. (see Page 7, Line 119)

9. Ethical considerations
   I cannot find any information concerning ethics despite the statement about ethical approval and information given to the participants found on p 12. Information about if informed consent was obtained, how data was handled and stored and how the participants’ identities have been protected are missing. Also information is lacking about the relationship between the researchers who recruited the participants and the researcher who conducted the interviews, and the participants. Were the researchers in any caregiving position for the participants? Can it be assumed that any power relations between the researchers and the participants existed so that the participants could feel forced to participate?

**RESPONSE:** We added the ethical information in the Methods section: “Informed consent was obtained from all participants before the interviews, and they were informed of their right to withdraw from the study at any time.” (see Page 7, Lines 122 - 124); “Data was handled confidentially, with all participant information de-identified using unique identification numbers. These identifiers were stored separately from the study data on a secure, password-protected drive. Only the principal investigator and select research staff had access to the encrypted file linking identification numbers to subject identities. The Office of Human Research was responsible for monitoring and auditing the collected data to ensure compliance with ethical standards.” (see Page 11, Lines 195 – 201); and “To minimize personal biases and influence, several steps were taken. YS and YM, who had no prior relationships or power dynamics with any participants, managed participant recruitment to ensure a diverse sample. Additionally, YS conducted all interviews in Mandarin, maintaining neutrality throughout the data collection process.” (see Page 9, Line 162 – 165).

10. Results
    In the method section only patients with a diagnosis of stroke are stated to be participants. I was therefore very surprised to understand from line 181-182 and the very first quotation in the results, that information given by relatives to the participants was included. If relative’s statements are to be part of data, then they also needs to be viewed as participants in the study.

**RESPONSE:** We apologize for the confusion. Since family caregivers only provided assistance to the participants (e.g., repeating their words when their pronunciation was difficult to understand), they were not considered participants in this study. We addressed this in the Methods section as follows: “Family caregivers were encouraged to accompany participants and provide needed assistance, especially for those with mild aphasia that could affect their communication. Their presence was solely for assistance purposes, and if any interference in the interview process was observed, the family caregivers were asked to leave to minimize their influence on the interview.” (see Page 10, Lines 185 – 189) To avoid confusion, we also removed the caregiver’s quote from the results section.

11. There are multiple problems related to the results, mostly pertaining to the headings of the themes and sub themes.
    The sub theme “Severity and complexity”, I do not see nether severity nor complexity in the text that describes what is labelled as this. Please revise.

**RESPONSE:** We revised the subtheme “Severity and complexity” to “Need for supervision and physical assistance.” (see Page 19, Line 239)

12. I am not very sure about the name of the second theme, line 208. Is the use of “survivor” really good? We can assume that no dead person will have the need of a caregiver of any kind. I suggest to use the word participant or the word stroke instead.

**RESPONSE:** Thank you for the suggestion. We revised Theme 2 to “The expectations of participants toward foreign caregivers.” (see Page 20, Line 268)

13. I have trouble to understand how anyone hiring a foreign caregiver can view language barrier as an “unexpected consequence”. Is really seen in the interviews or is this a result of a bad heading for the sub theme? Make sure that this section reports on the results, not discuss them.

**RESPONSE:** We apologize for any confusion caused by the original headings. We have revised the theme name to 'The challenges of employing a foreign caregiver' and changed the subtheme from 'Language barriers' to 'Communication difficulties.” (see Page 24, Line 326)

14. Discussion has a section dedicated to that. Examples that needs to be removed from the results are for example line 225.

**RESPONSE:** Thank you for the suggestion. We removed the redundant descriptions.

15. It is not ok to use valuing word in the results section, these should be reserved for the discussion, see for example first word, line 309.

**RESPONSE:** We removed the word “Unexpectedly” and made sure no other valuing word in the results section. (see Result section)

16. Discussion
    Well-written and problematizing the results in a good way. This is really well needed with this kind of problematic and bothering results showing so little respect for other human beings.

**RESPONSE:** Thank You.

17. Strength and limitations
    Discusses the limitations in a proper way. No strengths in the study is reported which I consider would be of value. There cannot only be limitations in the study, there must also exist some good things. I also do not agree that there would be greater reasons for persons with negative experiences to decline participation, than it would be for persons with positive experiences. Those who are dissatisfied are often not hesitant to tell about it.

**RESPONSE:** We have added the strengths of the study (see Page 32, Lines 477 – 485). Additionally, we have removed the limitation regarding individuals with negative experiences possibly declining to participate in the study, as per your suggestion (see Page 32, Line 486 – 493).

18. Conclusion
    Supported by the results.

**RESPONSE:** Thank You.

19. References
    Reference 19 – the title of the publication have been written in a mysterious way: Approaches QIaRDCAF. Similar problem appear in ref 33.
    The journal names are alternately written with initial upper-case and lower-case in each word. Make sure to adhere to the journals guidelines concerning this.

**RESPONSE:** We have corrected the errors. (See Pages 35 – 36, Ref. [25] and [42])

20. Language
    Good

**Reviewer #2:** Thank you for addressing an important phenomenon in caregiving for stroke patients in an Asian country. In Asian countries, reliance on informal caregivers is common due to the reluctance to institutionalise stroke patients in care homes, related to their local culture of filial piety. The current manuscript still contains many areas that require improvement.

I hope the authors can address the following issues:

1. The study is being presented as a phenomenological study. Please mention the key characteristics of a phenomenological study in the methods section. Otherwise, it appears that the study is more of a generic qualitative study. Important characteristics include the focus on lived experience, the meaning/interpretation of the phenomenon by the study participants, steps taken to bracket or to limit researcher bias/ influence in the interpretation fo the data etc

**RESPONSE:** Thank you for this critical suggestion. We revised the study design as a descriptive qualitative approach. (see Page 7, Line 119),

2. Secondly, the introduction needs to provide more information on the local context of the study. Were trained formal caregivers available in the country? How are foreign caregivers employed and what are the laws or restrictions surrounding the employment of these foreign helpers? Do they receive training prior to employment? What is the current long-term care system in Taiwan?

**RESPONSE:** We provided more detailed information on the local context of the study (see Page, Lines): “Since 1992, the Taiwanese government has introduced live-in foreign caregivers…more complex health needs [19].” (see Pages 4 – 5, Lines 79 – 94)

3. In the introduction section, add justification on why it is important to understand the perspectives of the stroke survivors. Currently it is only briefly and superficially described. What are the possible benefits of knowing their expectations, needs, challenges related to employing foreign caregivers? What expected policy changes are required?

**RESPONSE:** We revised this section as following: “Given the growing stroke population in Taiwan and the unique physical, cognitive, and emotional challenges resulting from stroke [24], it is important to gain insight into the caregiving experiences of this population. This qualitative study aimed to describe the lived experiences of older stroke survivors employing foreign caregivers in Taiwan to better understand their expectations, needs, and challenges. The findings will not only help improve care services provided by foreign caregivers but also assist policymakers in identifying the unmet needs of this population. Ultimately, this could enhance care quality, support caregivers in their roles, and guide policy recommendations for Taiwan’s long-term care system and other developed countries.”(see Page 6, Lines 106 – 115)

4. In the methods section, please include a paragraph to represent the positionality of the researchers in the study. This is important for phenomenological studies and is part of the COREQ criteria. What additional steps were taken to minimise personal bias / influence?

**RESPONSE:** We added the section “Positionality of the Research Team” in the Methods (see Pages 8 - 9, Lines 147 - 170).

5. Provide more description on the development of the topic guide. Was it based on theory or specific research questions? Please provide the full topic guide.

**RESPONSE:** We added the descriptions about the development of the interview guide in the Procedure Section: “To obtain an in-depth understanding of the stroke survivors receiving care from foreign caregivers, the research team developed a semi-structured interview guide based on an extensive literature review addressing our research questions. This guide included seven major open-ended questions, which were reviewed by an interdisciplinary expert panel, as well as stroke survivors, and caregivers. The full interview guide is available in **S2 Table**, with examples of key questions provided below…” (see Pages 9 – 10, Lines 172 – 176 and **S2 Table** for the full interview guide).

6. What language was used to conduct the interview? What language was used for transcription and analysis?

RESPONSE: We apologize for the confusion. We used Mandarin to conduct the interview, transcription and analysis, and then translated the coded results into English. We explained this process in the Procedure and Data Analysis sections: “YS conducted in-depth, face-to-face interviews with participants in Mandarin.” (see Page 10, Lines 182 – 183) “Two Taiwanese researchers (YS and FC) independently read, abstracted, and coded the transcripts immediately after data collection. The coded data and quotes were then translated from Mandarin to English by YS and FC, with accuracy verified by a bilingual team member (YL). Data with similar meanings were grouped and synthesized into descriptive themes, which were reviewed by the entire research team [34]. Analysis continued until thematic saturation was reached, with co-coders agreeing that no new categories emerged [34, 35]. The translated coding results were reviewed and discussed until consensus was reached on the final interpretation of participants’ experiences [35, 36].” (see Pages 11 - 12, Lines 205 – 214).

7. Line 140-141: How does presence of family caregivers influence the interview?

**RESPONSE:** Since family caregivers only provided assistance to the participants (e.g., repeating their words when their pronunciation was difficult to understand), their influence on the interview was expected to be minimal. We added the description as following: “Family caregivers were encouraged to accompany participants and provide needed assistance, especially for those with mild aphasia or cognitive impairments that could affect their communication. Their presence was solely for assistance purposes, and if any interference in the interview process was observed, the family caregivers were asked to leave to minimize their influence on the interview.” (see Page 10, Lines 185 – 189)

8. Lines 152: Provide an explanation for textural descriptions with a reference. It is good to provide a sample of the coding process.

**RESPONSE:** We revised the sentences in the Data analysis section: “Thematic analysis was performed to interpret the meaning of textual data [33]. Two Taiwanese researchers (YS and FC) independently read, abstracted, and coded the transcripts immediately after data collection. The coded data and quotes were then translated from Mandarin to English by YS and FC, with accuracy verified by a bilingual team member (YL). Data with similar meanings were grouped and synthesized into descriptive themes, which were reviewed by the entire research team [34]. For example, quotes that were coded as “obedient” and “respectful” were grouped under the subtheme “Being obedient and submissive.” (see Pages 11 – 12, Lines 204 – 211)

9. Line 153: usually the term used is "thematic saturation"

**RESPONSE:** We revised the sentence as following: “Analysis continued until thematic saturation was reached, with co-coders agreeing that no new categories emerged [34, 35].” (see Page 12, Lines 211 – 212)

10. A total of 23 stroke survivors were recruited for the study. How did the researchers determine when recruitment would be terminated?

**RESPONSE:** Please see the Study Setting and Recruitment section: “Recruitment continued on a rolling basis until data saturation was reached, with no new information emerging from interviews [30].” (see Page 7, Lines 134 – 135) and the Results section: “Data saturation was reached after interviewing the 20th participant, with the research team interviewing three additional participants to confirm that no new themes was emerging. Recruitment concluded after the 23rd interview, as no further insights were identified.” (see Page 12, Lines 224 – 227)

11. For the results section, phenomenological studies usually provide rich description which can illustrate the lived experience.The current results section seem to be inadequate. For example, under Severity or complexity, the provided quotes are inadequate to reflect the severity or complexity of the patient's condition.

**RESPONSE:** We revised the study design to a descriptive qualitative study, as suggested by Reviewer 1. (see Page 7, Lines 119)

12. Overall, the results section show a more superficial description of the study findings rather than providing an interpretation of the lived experience of the stroke survivors.

**RESPONSE:** We apologize for the confusion. As noted earlier, we have revised the study design to a descriptive qualitative study in accordance with Reviewer 1’s suggestion. (see Page 7, Lines 119)

13. The discussion provided a good explanation of the cultural setting of expectations towards foreign caregivers. The authors could provide some conclusion on the ethics or appropriateness of these expectations. Provide some recommendations on policy or education for potential employers of foreign caregivers.

**RESPONSE:** Thank you for your feedback. We have expanded the Discussion and Conclusion sections to address the ethical considerations surrounding the survivors' expectations, as well as to provide recommendations on policy and education for the employment of foreign caregivers. (see Pages 28 – 34, Lines 401 – 514)

14. Please complete the COREQ checklist and upload it as a supplementary information for this study. Thank you.

**RESPONSE:** We have completed the COREQ checklist and uploaded the as a supplementary file (see S1 File).

1.	Munkejord MC, Ness TM, Silan W. ‘We are all interdependent’. a study of relationships between migrant live-in carers and employers in Taiwan. Glob Qual Nurs Res. 2021;8:23333936211043504.

2.	Porat I, Iecovich E. Relationships between elderly care recipients and their migrant live-in home care workers in Israel. Home Health Care Services Quarterly. 2010;29(1):1-21.

3.	Salami B, Duggleby W, Rajani F. The perspective of employers/families and care recipients of migrant live-in caregivers: a scoping review. Health & Social Care in the Community. 2017;25(6):1667-78.

4.	Tseng SH, Chang FH. Transitioning from hospitals to the community: perspectives of rehabilitation patients with neurological disorders and their service providers. Disabil Rehabil. 2017;39(23):2420-7.
