# Supplementary material for: Employing foreign caregivers: A qualitative study of the perspectives of older stroke survivors
Source: PLoS One. 2025 Jan 3;20(1):e0316757. doi: 10.1371/journal.pone.0316757 (PMC11698342; doi:10.1371/journal.pone.0316757)
Supplement: Attachment — Submitted filename: Response to Reviewers.docx [file pone.0316757.s005.docx]

PONE-D-24-31334R1

Employing foreign caregivers: exploring perspectives of older stroke survivors - a qualitative study

PLOS ONE

Reviewers' comments:

Reviewer's Responses to Questions

**Comments to the Author**

1. If the authors have adequately addressed your comments raised in a previous round of review and you feel that this manuscript is now acceptable for publication, you may indicate that here to bypass the “Comments to the Author” section, enter your conflict of interest statement in the “Confidential to Editor” section, and submit your "Accept" recommendation.

Reviewer #1: All comments have been addressed

Reviewer #2: (No Response)

2. Is the manuscript technically sound, and do the data support the conclusions?

The manuscript must describe a technically sound piece of scientific research with data that supports the conclusions. Experiments must have been conducted rigorously, with appropriate controls, replication, and sample sizes. The conclusions must be drawn appropriately based on the data presented.

Reviewer #1: (No Response)

Reviewer #2: Yes

3. Has the statistical analysis been performed appropriately and rigorously?

Reviewer #1: (No Response)

Reviewer #2: N/A

4. Have the authors made all data underlying the findings in their manuscript fully available?

The [PLOS Data policy](http://www.plosone.org/static/policies.action#sharing) requires authors to make all data underlying the findings described in their manuscript fully available without restriction, with rare exception (please refer to the Data Availability Statement in the manuscript PDF file). The data should be provided as part of the manuscript or its supporting information, or deposited to a public repository. For example, in addition to summary statistics, the data points behind means, medians and variance measures should be available. If there are restrictions on publicly sharing data—e.g. participant privacy or use of data from a third party—those must be specified.

Reviewer #1: (No Response)

Reviewer #2: Yes

5. Is the manuscript presented in an intelligible fashion and written in standard English?

PLOS ONE does not copyedit accepted manuscripts, so the language in submitted articles must be clear, correct, and unambiguous. Any typographical or grammatical errors should be corrected at revision, so please note any specific errors here.

Reviewer #1: (No Response)

Reviewer #2: No

6. Review Comments to the Author

Please use the space provided to explain your answers to the questions above. You may also include additional comments for the author, including concerns about dual publication, research ethics, or publication ethics. (Please upload your review as an attachment if it exceeds 20,000 characters)

Reviewer #1: (No Response)

Reviewer #2: Thank you for addressing most of the comments in the first round of review.
There are still some minor language issues that require revision.

Introduction, line 65: instead of saying "a high number of family caregivers are needed", it would be more appropriate to state that "the caregivers' role is crucial". It is not so much the number of family caregivers, but the availability and quality of care by the caregivers that enables stroke survivors to return to the community safely.

**Response:** Thank you for the critical feedback. We have revised this sentence to “Family caregivers are essential for reducing this burden and assisting stroke survivors in returning to their homes and communities.” (See **page 5, lines 73-75**)

Methods, Design, line 119-120: Please correct the lanugage ".. to understand stroke survivors' experiences in receiving care from..."

**Response:** We have revised this sentence accordingly. (See **page 9, lines 144-145**)

Methods, Design, line 124: Please remove the Joanna Briggs Institute Critical Appraisal Checklist from the text. A critical appraisal checklist is meant for evaluating quality of published studies and not to guide reporting. The COREQ checklist is adequate.

**Response:** We have removed the Joanna Briggs Institute Critical Appraisal Checklist and only kept the COREQ checklist. (See **page 9, lines 150-151**)

Methods, Study Setting and Recruitment, line 132-133: Please describe how you approached potential participants. Were they approached at the waiting area of the rehabilitation services or were they recruited through an advertisement or poster? Who did the recruitment? Were the interviews conducted on the spot or an appointment given for a protected time for the interview?

**Response:** We described how we approached the potential participants at **pages 9-10, lines 160-165**): “Patients with stroke were screened for study inclusion eligibility by staff at the rehabilitation clinics, and potentially eligible patients were introduced to the study researchers, who then met with the patients in person and explained the purpose of the study by using informational leaflets.”

We also described the place where we conducted the interviews at **page 13, lines 220-222**: The interviews were scheduled at times that were convenient for the participants and took place in quiet settings, either at academic medical centers or in the participants’ homes.

Study participants, line 140: please replace "Chinese" with "Mandarin" to standardise the manuscript (as mentioned in line 183).

**Response:** We have replaced "Chinese" with "Mandarin" accordingly. (See **page 10, line 173**)

Formatting of quotes, e.g. line 281-283, lines 309-310, lines 343-347, lines 357-358, lines 380-389, lines 394-397. Please separate the quotes from different participants into separate paragraphs.

**Response:** We have separated the quotes from different participants into separate paragraphs. (See the **Results** section **pages 22-33**)

Discussion, lines 470-473. Please highlight the issue of patient safety when untrained caregivers are used, particularly when one of the quotes mentioned that hte caregiver caused the participant to fall. What does "inadequately treated" mean? Please rephrase for clarity.

**Response:** We revised this paragraph and highlighted the issue of patient safety as following: “Training is essential for safety. One participant in the current study recounted an incident in which his caregiver’s inexperience caused him to fall early in their working relationship. Some participants also reported feeling neglected or disrespected. Others did not receive adequate attention from their foreign caregivers.” (See **page 37, lines 575-579**)

References, please ensure references are correctly formatted. Some were incomplete e.g. ref 19, and others used different referencing styles. Referencing for websites should include when the website was accessed.
**Response:** Thank you for pointing out this issue. We have thoroughly reviewed all references and ensured their accuracy and consistency with the required formatting style. Additionally, for website references, we have included the date of access as recommended.

I suggest that the manuscript undergo professional proofreading prior to resubmission.

**Response:** Thank you for your suggestion. We have sent the manuscript for professional proofreading, and the necessary edits have been made to ensure clarity and accuracy.
